# Vasopressin Effectively Suppresses Male Fertility

**DOI:** 10.1371/journal.pone.0054192

**Published:** 2013-01-24

**Authors:** Woo-Sung Kwon, Yoo-Jin Park, Yun-Hee Kim, Young-Ah You, In Cheul Kim, Myung-Geol Pang

**Affiliations:** 1 Department of Animal Science & Technology and BET Research Institute, School of Bioresource & Bioscience, Chung-Ang University, Anseong, Gyeonggi-do, Korea; 2 National Institute of Animal Science, Rural Development Administration, Cheonan, Chungcheongnam-do, Korea; National Cancer Institute, United States of America

## Abstract

Arginine vasopressin (VP) is neurohypophysial hormone has been implicated in stimulating contractile activity of the male reproductive tract in the testis. Higher levels of VP decrease sperm count and motility. However, very little is known about the involvement of VP in controlling mammalian reproductive process. The goal of this study was to confirm that effect of VP receptor (AVPR2) on sperm function in capacitation condition. Deamino [Cys 1, D-ArgS] vasopressin (dDAVP), an AVPR2 agonist that operates only on AVPR2, was used. Also, Mouse spermatozoa were incubated with various concentrations of dDAVP (10^−11^–10^−5^ M) and sperm motility, capacitation status, Protein Kinase A activity (PKA), tyrosine phosphorylation, fertilization, and embryo development were assessed using computer-assisted sperm analysis, Combined Hoechst 33258/chlortetracycline fluorescence, Western blotting, and *in vitro* fertilization, respectively. AVPR2 was placed on the acrosome region and mid-piece in cauda epididymal spermatozoa, but the caput epididymal spermatozoa was mid-piece only. The high dDAVP treatment (10^−8^ and 10^−5^ M) significantly decreased sperm motility, intracellular pH and PKA substrates (approximately 55 and 22 kDa) and increased Ca^2+^ concentration. The highest concentration treatment significantly decreased PKA substrate (approximately 23 kDa) and tyrosine phosphorylation (approximately 30 kDa). VP detrimentally affected capacitation, acrosome reaction, and embryo development. Treatment with the lowest concentration (10^−11^ M) was not significantly different. Our data have shown that VP stimulates ion transport across sperm membrane through interactions with AVPR2. VP has a detrimental effect in sperm function, fertilization, and embryonic development, suggesting its critical role in the acquisition of fertilizing ability of mouse spermatozoa. These research findings will enable further study to determine molecular mechanism associated with fertility in capacitation and fertilization. It is also an important pivotal precondition to the progress of diagnostic test to identify infertility and to apply male contraception.

## Introduction

Arginine vasopressin (hereafter referred to as vasopressin, VP, and also known as antidiuretic hormone), which is found in most mammals including humans, is essential for cardiovascular homeostasis (i.e., survival) [1]. Besides its principle role, this neurohypophysial hormone has been implicated in stimulating contractile activity of the male reproductive tract, with each including dose-dependent seminiferous tubule contraction in the testis [2], [3]. High concentrations of immunoreactive VP and specific vasopressin receptor have been found in the testis [3], [4], [5], [6].

VP increases the volume of seminal fluid and the concentration of spermatozoa in the ejaculate of the rabbit [7]. VP increases the accumulation of cytosolic cAMP in vas deferens epithelial cells and can modulate ion transport across vas deferens epithelia by independent mechanisms [8].

However, very little is known about the involvement of VP in controlling the mammalian reproductive process. Puri and Puri [9] discovered that a higher level of VP decreases the sperm count and motility in healthy human but infertile men. More recently, Sliwa [10] reported that VP plays a role in the decreased sperm motility in mouse vas deference. Intriguingly, Oh [11] discovered that VP receptor 2 (arginine vasopressin receptor 2; AVPR2) was differentially expressed between small and large litter size group spermatozoa in porcine. AVPR2 from small litter size group spermatozoa was increased more so than the expression level of the large one. Therefore, the association between VP and fertility is a continuing source of concern.

The present study was designed to explore the expression of VP receptor in spermatozoa and the roles of VP in sperm function, fertilization, and embryonic development.

## Materials and Methods

All procedures were performed according to guidelines for the ethical treatment of animals and approved by Institutional Animal Care and Use Committee in Chung-Ang University, Seoul, Korea.

### Media and reagents

All components were purchased from Sigma-Aldrich (St Louis, MO, USA). Modified Tyrode's medium [12] was used as basic medium (BM) (97.84 mM NaCl, 1.42 mM KCl, 0.47 mM MgCl_2_·H_2_O, 0.36 mM NaH_2_PO_4_·H_2_O, 5.56 mM D-glucose, 25 mM NaHCO_3_, 1.78 mM CaCl_2_·H_2_O, 24.9 mM Na-lactate, 0.47 mM Na-pyruvate, and 50 µg/ml gentamycin). The medium was incubated before the day of experiment. Bovine serum albumin (BSA; 4 mg/ml) was added to the BM for sperm capacitation. A stock solution (10^−3^ M) of the AVPR2 agonist deamino [Cys 1, D-ArgS] vasopressin (dDAVP) [13], [14] was diluted with BM and stored at −20°C. For treatment stock solution was added to the BM to final molar concentration of 10^−11^, 10^−8^, and 10^−5^ M.

### Sperm preparation and treatment

Mouse sperm suspension was prepared using 8–12 week-old male ICR mice (Nara Biotech, Seoul, Korea). The caput and cauda epididymides from mice were separated, the fat was removed, and the fat-free samples were placed in BM containing 0.4% BSA in 35 mm-diameter sterile cell culture dishes. Caput or cauda epididymides were cut with a surgical blade. The excised cauda epididymidis minced a few pieces to let spermatozoa flow out from the ducts [15]. The spermatozoa from the cauda epididymis were incubated to disperse for 10 min at 37°C in an atmosphere containing 5% CO_2_. The caput epididymis sperm suspension was layered in a 15 ml Falcon tube on a discontinuous 90% and 45% Percoll gradient in 90% Pecoll ∶ 45% Pecoll ∶ sperm suspension (1 ∶ 1 ∶ 1) and centrifuged at 500× g for 13 min. The top two layers were carefully separated and then last layer washed two times with 1 ml BM. The cauda epididymis sperm suspension was incubated for 90 min at 37°C in 5% CO_2_ in air for capacitation in BM containing 0.4% BSA that was additionally supplemented with 10^−11^, 10^−8^, and 10^−5^ M dDAVP.

### Immunolocalization of AVPR2

The dispersed spermatozoa from the tissue were suspended in Dulbecco's phosphate buffered saline (DPBS), placed on a glass slide, and allowed to air-dry [16]. The sperm were then fixed with 3.7% paraformaldehyde for 30 min at 4°C. After fixation, the cells were washed with DPBS containing 0.1% Tween-20 (PBST) and blocked in 5% BSA in PBST for 1 h at 4°C. The slide was incubated with rabbit polyclonal to AVPR2 IgG primary antibody (Abcam, Cambridge, UK) diluted with 5% BSA in PBST (1∶50), lectin Peanut agglutinin (PNA) [17], [18] conjugated with Alexa Fluor 647 (Molecular Probes, Carlsbad, CA, USA) diluted with 5% BSA in PBST (1∶100) overnight at 4°C. Slides were washed two times in PBST and DPBS, and then incubated for 2 h at room temperature (RT) with fluorescein isothiocynate (FITC)-conjugated goat polyclonal rabbit IgG secondary antibody (Abcam, Cambridge, UK) diluted with 5% BSA in PBST 1∶100. The spermatozoa were counterstained with 4′,6-diamidino-2-phenylindole (DAPI). The results were observed using a model TS-1000 microscope equipped with NIS Elements image software (Nikon, Tokyo, Japan).

### Computer-assisted sperm analysis (CASA)

The sperm motility (%) was measured using a CASA system (SAIS plus version 10.1; Medical Supply, Seoul, Korea). Briefly, 10 µl of sample was placed in a Makler chamber (Makler, Haifa, Israel). The filled chamber was placed on a 37°C stage. Using a 10× phase contrast objective, the image was relayed, digitized, and analyzed by the SAIS software. The movement of at least 250 sperm cells was recorded for each sample from more than five randomly selected fields. The program used for user-defined settings were: frames acquired, 20; frame rate, 30 Hz; minimum contrast, 7; minimum size, 5; low/high size gates, 0.4–1.5; low/high intensity gates, 0.4–1.5; non-motile head size, 16; non-motile brightness, 14.

### Combined Hoechst 33258/chlortetracycline fluorescence assessment of capacitation status (H33258/CTC)

Capacitation status was determined by the dual staining method described by Pérez et al. [19] with some modifications. Briefly, 135 µl of treated spermatozoa were added to 15 µl of H33258 solution (10 µg H33258/ml DPBS) and incubated for 2 min at RT. Excess dye was removed by layering the mixture over 250 µl of 2% (w/v) polyvinylpyrrolidone in DPBS. After centrifuging at 100× g for 2.5 min, the supernatant was discarded and the pellet resuspended in 100 µl of DPBS; 100 µl of a freshly prepared chlortetracycline fluorescence (CTC) solution (750 mM CTC in 5 µl buffer: 20 mM Tris, 130 mM NaCl, and 5 mM cysteine, pH 7.4). Samples were observed with a Microphot-FXA microscope (Nikon) under epifluorescence illumination using ultraviolet BP 340–380/LP 425 and BP 450–490/LP 515 excitation/emission filters for H33258 and CTC, respectively. The spermatozoa were classified as live non-capacitated (F type, bright green fluorescence distributed uniformly over entire sperm head, with or without stronger fluorescent line at equatorial segment), live capacitated (B type, green fluorescence over acrosomal region and a dark post acrosome), or live acrosome reacted (AR type, sperm showing a mottled green fluorescence over head, green fluorescence only in post acrosomal region or no fluorescence over the head) [20]. All spermatozoa had bright green fluorescent mid-pieces. Two slides per sample were evaluated with at least 400 spermatozoa per slide.

### Measurement of intracellular calcium ion concentration ([Ca^2+^]_i_)

5 µM Fura-2 acetoxymethyl ester (AM) (Molecular Probes) was added 60 min after the onset of capacitation [21]. The dye was allowed to load passively into cells for the remaining 30 min of capacitation at 37°C in an atmosphere of 5% CO_2_ in air. At the end of the loading period, samples were washed once by centrifugation at 200× g for 10 min and then resuspended in DPBS. The sperm suspension was illuminated with two excitation wavelengths (340 nm and 380 nm) and the emitted fluorescence was measured at 510 nm [22], [23], [24]. [Ca^2+^]_i_ was present as the ratio of fluorescence from excitation at 340 nm to that at 380 nm (F340/F380) [23], [25]. The ratio of fluorescence was detected with microplate fluorometer (Gemini Em; Molecular Devices Corporation, Sunnyvale, CA, USA) and calculated with SoftMax Pro 5 (Molecular Devices). All treatments were calculated ratio (340/380) and fluorescence ratio were calculated by ratio of dDAVP treatment/ratio of control, respectively.

### Measurement of intracellular pH ([pH]_i_)

5 µg/ml 2′,7′-bis-(2-carboxyethyl)-5-(and-6)-carboxyfluorescein AM (Molecular Probes) was added 60 min after the onset of capacitation. The dye was allowed to load passively into cells for the remaining 30 min of capacitation at 37°C in an atmosphere of 5% CO_2_ in air. After capacitation, each treated sample was washed once by centrifugation at 200× g for 10 min. Dye-loaded samples were resuspended in DPBS. For calibration based on nigericin, 0.5 ml of high K^+^ calibration buffer received 5 µl of the cell suspension and 1 µl of nigericin (Molecular Probes) and the mixture was incubated for 20 min at RT. High K^+^ buffer consisted 140 mM KCl, 1 mM MgCl_2_, 2 mM CaCl_2_, 5 mM glucose, and 20 mM MES or 20 mM Tris. Several pH ranges were titrated by mixing appropriate amounts of MES-buffered (acidic) or Tris-buffered (basic) solution. Nigericin was made up at 1 mg/ml absolute ethanol and stored at −20°C. The fluorescence was detected with a Gemini Em microplate fluorometer (Molecular Devices Corporation) and calculated with SoftMax Pro 5 software (Molecular Devices Corporation).

### Western blot analysis of sperm proteins

To quantify AVPR2, Phospho-Protein Kinase A (PKA) substrate and tyrosine phosphorylation protein from mouse spermatozoa, Western blotting was performed as described previously [26] with some modifications. Aliquots of dDAVP treated with medium (0.4% BSA) spermatozoa were centrifuged at 10,000× g for 5 min and washed twice with DPBS. Sperm pellets were resuspended in sample buffer [26] containing 5% 2-mercaptoethanol and incubated for 10 min at RT. After incubation, the soluble and insoluble fractions were separated by centrifugation at 10,000× g for 10 min and the supernatants were saved. Samples were subjected to SDS-polyacrylamide gel electrophoresis using a 12% mini-gel system (Amersham, Piscataway, NJ, USA) and the separated proteins were transferred to a polyvinylidene fluoride membrane (Amersham). The membrane was blocked with 3% blocking agent (Amersham, Piscataway, NJ, USA) for 1 h at RT. Phospho-PKA substrates from dDAVP treated spermatozoa was immunodetected by anti-phospho-PKA substrate rabbit polyclonal antibody (Cell Signaling Technology, Danvers, MA, USA) diluted with blocking solution (1∶10,000) overnight at 4°C. Next the membrane incubated with a horse-radish peroxidase (HRP) conjugated Goat Anti-Rabbit IgG (Abcam) diluted with blocking solution (1∶5,000) for 1 h at RT. Tyrosine phosphorylation proteins from dDAVP treated spermatozoa was immunodetected by a HRP conjugated monoclonal anti-phosphotyrosine mouse antibody (PY20; Abcam) diluted with blocking solution (1∶2,500) overnight at 4°C. And α-tubulin was detected by incubation with monoclonal anti α-tubulin mouse antibody (Abcam) diluted with blocking solution (1∶10,000) for 2 h at RT. Also, membranes were incubated with a HRP conjugated Goat Anti-Mouse IgG (Abcam) diluted with blocking solution (1∶10,000) for 1 h at RT. Membranes was washed three times with PBST. The proteins on the membrane were visualized with an enhanced chemiluminescence (ECL) technique using ECL reagents. All bands were scanned with GS-800 calibrated imaging densitometer (Bio-Rad, Hercules, CA, USA) and analyzed with Quantity One (Bio-Rad). Finally, bands were calculated by ratio of Phospo-PKA substrate/α-tublin or tyrosine phosphorylated protein/α-tublin.

### *In vitro* fertilization

Eight to twelve week-old female B6D2F1/CrljOri hybid mice were purchased from Nara Biotech (Seoul, Korea). These mice were superovulated with 5 IU of pregnant mare serum gonadotrophin given i.p., and 5 IU of human chorionic gonadotrophin given i.p. 48 h later. Fifteen hours after the second injection, the female mice were sacrificed and Cumulus-oocyte complexes (COCs) were collected from oviduct with DPBS in sterile culture dish. The COCs were placed within 50 µl of BM supplemented with 10% FBS under mineral oil and then incubated for 1 h before insemination at 37°C in an atmosphere of 5% CO_2_ in air. After capacitation, treated spermatozoa were washed with BM supplemented with 0.4% BSA and the 1×10^6^/ml spermatozoa were gently inseminated into the incubated oocytes and incubated for 6 h at 37°C in an atmosphere of 5% CO_2_ in air. After fertilization only normal embryos were collected and moved other new drop and then incubated in 50 µl of BM supplemented with 0.4% BSA. After 18 h from insemination point, fertilization rate was assessed by determining the number of two-cell embryos. All two-cell embryos were grouped and cultured in a new drop (50 µl) of BM supplemented with 0.4% BSA for 5 days at 37°C in an atmosphere of 5% CO_2_ in air. All embryos that developed up to the blastocyst stage were counted.

### Statistical analysis

The data were analyzed using one-way ANOVA of SPSS (v. 12.0; Chicago, IL, USA), with a Tukey's test to locate differences. This test compares responses within replicates; for a significant difference to be obtained, a consistent and reasonable magnitude is required between control and treated sample. *p*<0.05 was considered statistically significant and data are expressed as mean ± SEM.

## Results

### Immunolocalization of AVPR2 in the spermatozoa from caput and cauda epidydimis

**Figure 1:**
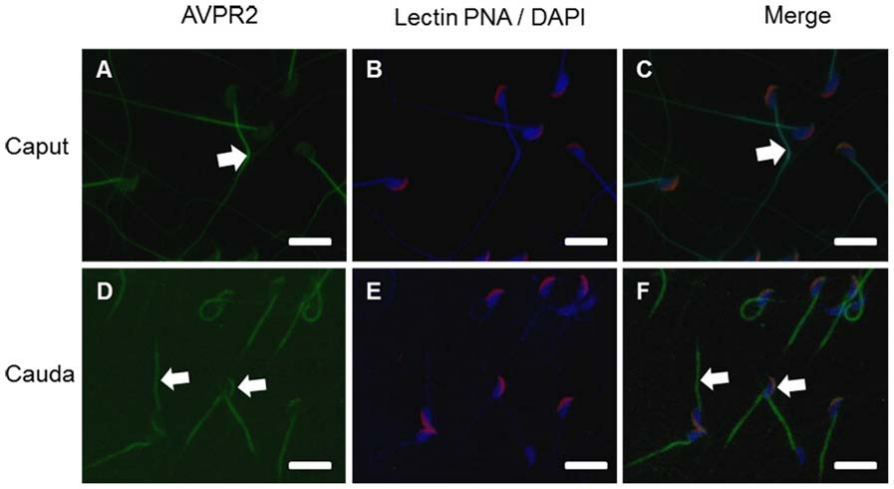
Localization of AVPR2 in epididymal mouse spermatozoa. Immunohistochemistry staining of mouse caput (A, B, C) and cauda (D, E, F) spermatozoa. B and E are merged images of acrosome (Lectin PNA conjugated Alex 647, red) and nucleus (DAPI, blue). C and F are merged image of AVPR2, acrosome and nucleus. Arrows directed AVPR2 (A, C, D, and F). Images obtained using Nikon TS-1000 microscope with NIS Elements image software (Nikon, Japan). Bar = 10 µm.

Mouse spermatozoa from caput and cauda epidydimis were examined by immunofluorescence using antibody AVPR2, lectin PNA, and DAPI (Fig. 1). Caput spermatozoa had intense AVPR2 signals on the mid-piece while the other cellular regions were unlabeled (Fig. 1A, arrow). Caput sperm had detectable lectin PNA signals on the acrosome region (Fig. 1B). In contrast, most of cauda spermatozoa stained AVPR2 in both the acrosome and the mid-piece, also lectin PNA stained in the acrosome (Figs. 1D–F).

### Effect of dDAVP on sperm motility

**Figure 2:**
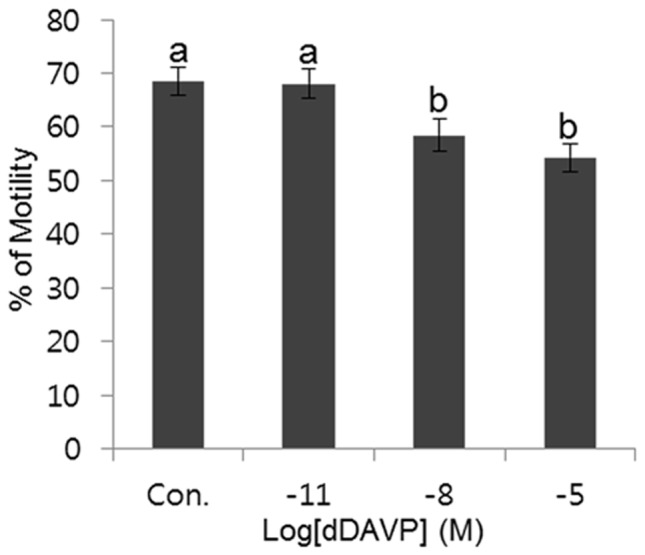
Change of the percentage of motility in various VP treatment concentrations. Data represent mean ± SEM, n = 6. Values with different superscripts (^a,b^) were significantly different between control and treatment groups by one-way ANOVA (p<0.05).

After 90-min incubation, the percentage of sperm motility were 68.53±2.72% in control, 68.08±2.66% in 10^−11^ M, 58.44±2.94% in 10^−8^ M, and 54.19±2.67% in 10^−5^ M dDAVP. Each treatment group of dDAVP displayed decreased motility (%) after incubation under capacitation condition. The percentage of motility was affected by dDAVP in a dose-dependent manner, however significantly decreased motility was observed only in the highest concentration (10^−5^ M) (*p*<0.05) (Fig. 2).

### Effect of dDAVP on change of [pH]_i_ and [Ca^2+^]_i_

**Figure 3:**
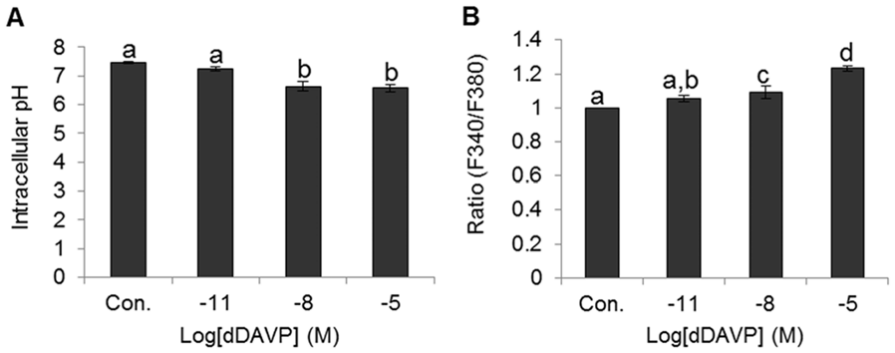
Change of [pH]_i_ and calcium concentration during incubation with dDAVP. (A) Change of [pH]_i_ in various treatment condition. (B) Change of [Ca^2+^]_i_ concentration in various treatment condition. Data represent mean ± SEM, n = 4. Values with different superscripts (^a,b,c,d^) were significantly different between control and treatment groups by one-way ANOVA (p<0.05).

[pH]_i_ was decreased by dDAVP in a dose-dependent manner. [pH]_i_ were 7.47±0.03 in control, 7.25±0.07 in 10^−11^ M, 6.64±0.16 in 10^−8^ M, and 6.58±0.13 in 10^−5^ M dDAVP. Significantly decreased [pH]_i_ compared with control and 10^−11^ M was found in high concentration (10^−8^ and 10^−5^ M) were (*p*<0.05) (Fig. 3A). The [Ca^2+^]_i_ was expressed as the ratio of the control. The [Ca^2+^]_i_ were 1.06±0.02 in 10^−11^ M, 1.09±0.04 in 10^−8^ M, and 1.23±0.02 in 10^−5^ M dDAVP. [Ca^2+^]_i_ was increased by dDAVP in a dose-dependent manner. Significantly increased [Ca^2+^]_i_ was observed in high concentrations (10^−8^ and 10^−5^ M) compared with control and 10^−11^ M (*p*<0.05) (Fig. 3B).

### Effect of dDAVP on the sperm capacitation status

**Figure 4:**
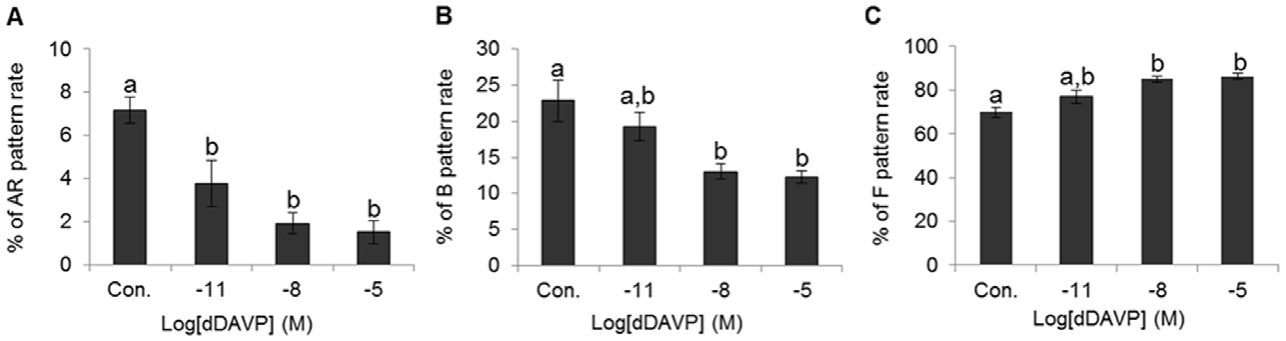
Effect of dDVAP on sperm capacitation status. (A) Change of acrosome reacted spermatozoa in various treatment condition. (B) Change of capacitated spermatozoa in various treatment conditions. (C) Change of non-capacitated spermatozoa in various treatment conditions. Data represent mean ± SEM, n = 3. Values with different superscripts (^a,b^) were significantly different between control and treatment groups by one-way ANOVA (p<0.05).

Decreased acrosome reaction and capacitation patterns were found in all treatment with dDAVP in dose-dependent manners. However non-capacitated spermatozoa were increased by dDAVP in a dose-dependent manner. The percentage of acrosome reaction were 7.17±0.61% in control, 3.79±1.08% in 10^−11^ M, 1.93±0.49% in 10^−8^ M, and 1.53±0.54% in 10^−5^ M dDAVP. The percentage of capacitated spermatozoa were 22.89±2.87% in control, 19.31±1.98% in 10^−11^ M, 13.03±1.08% in 10^−8^ M, and 12.3±0.82% in 10^−5^ M dDAVP. Also, the percentage of non-capacitated spermatozoa were 69.95±2.33 in control, 76.91±2.79% in 10^−11^ M, 85.04±1.54% in 10^−8^ M, and 86.17±1.37% in 10^−5^ M dDAVP. All dDAVP treatments displayed significantly decreased acrosome reaction compared with control (*p*<0.05) (Fig. 4A). Also, high concentration dDAVP treatments (10^−8^ and 10^−5^ M) produced significantly decreased capacitated spermatozoa and increased non-capacitated spermatozoa compared with control (Fig. 4B and 4C).

### Identification of protein kinase A substrates and protein tyrosine

**Figure 5:**
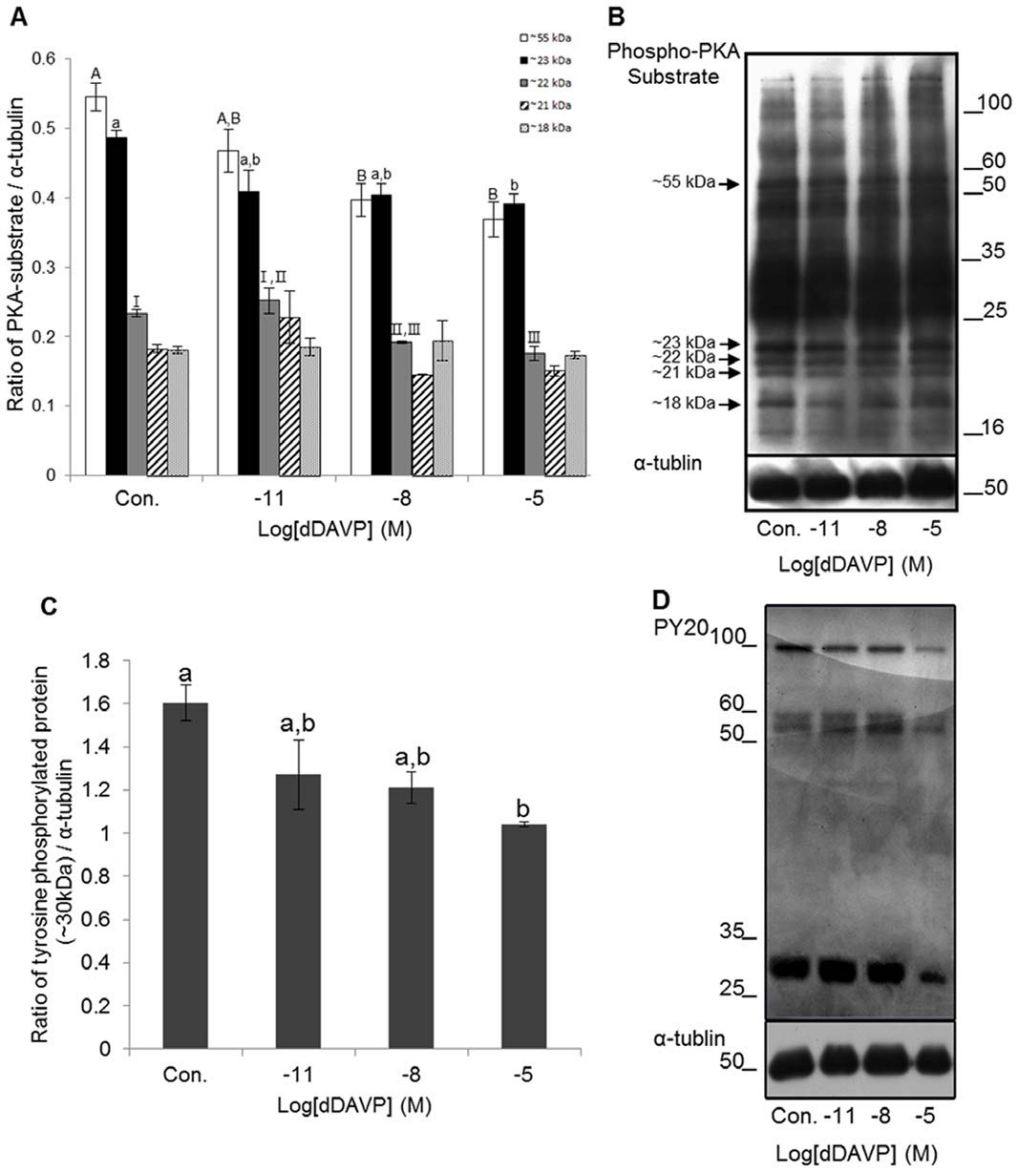
Effect of dDAVP on PKA activity and protein tyrosine phosphorylation. (A) Density of PKA substrates in various treatments (Open Bar: ∼55 kDa, Black Bar: ∼23 kDa, Grey Bar: ∼22 kDa, Stripe Bar: ∼21 kDa, and Dot Bar: ∼18 kDa). Data represent mean ± SEM, n = 3. Values with different superscripts (^A,B,a,b,I,II,III^) were significantly different between control and treatment groups by One-way ANOVA (p<0.05). (B) Phospho-PKA substrates were probed with anti-phospho-PKA substrates; lane 1: Control, lane 2: 10^−11^ M dDAVP, lane 3: 10^−8^ M dDAVP, lane 4: 10^−5^ M dDAVP. (C) Density of tyrosine phosphorylated protein (∼30 kDa) in various treatments. Data represent mean ± SEM, n = 3. Values with different superscripts (^a,b^) were significantly different between control and treatment groups by One-way ANOVA (p<0.05). (D) tyrosine phosphorylated proteins were probed with PY 20; lane 1: Control, lane 2: 10^−11^ M dDAVP, lane 3: 10^−8^ M dDAVP, lane 4: 10^−5^ M dDAVP.

Five different bands (approximately 55, 23, 22, 21 and 18 kDa) were confirmed from mouse spermatozoa after treatments (Fig. 5B). Phospho-PKA substrates were normalized with α-tublin. The 55 kDa Phospho-PKA substrates were 0.55±0.02 in control, 0.47±0.03 in 10^−11^ M, 0.4±0.02 in 10^−8^ M, and 0.37±0.03 in 10^−5^ M dDAVP. The 23 kDa Phospho-PKA substrates were 0.49±0.01 in control, 0.41±0.03 in 10^−11^ M, 0.4±0.02 in 10^−8^ M, and 0.39±0.02 in 10^−5^ M dDAVP. The 22 kDa Phospho-PKA substrates were 0.23±0.005 in control, 0.25±0.02 in 10^−11^ M, 0.19±0.001 in 10^−8^ M, and 0.18±0.01 in 10^−5^ M dDAVP. The Phospho-PKA substrates of 10^−8^ and 10^−5^ M in 55 kDa were significantly decreased compared with control (*p*<0.05). The Phospho-PKA substrate of 10^−5^ M in 23 kDa was significantly decreased compared with control (*p*<0.05). The Phospho-PKA substrates of 10^−8^ and 10^−5^ M in 22 kDa were significantly decreased compared with control (*p*<0.05). Also, the Phospho-PKA substrate of 10^−5^ M in 22 kDa was significantly decreased compared with 10^−11^ M (*p*<0.05) (Fig. 5A and 5B). Four different bands (approximately 100, 60, 55, and 30 kDa) were confirmed from mouse spermatozoa after treatments (Fig. 5D). Tyrosine phosphorylated proteins were normalized with α-tublin. The 30 kDa tyrosine phosphorylated proteins were 1.6±0.08 in control, 1.27±0.16 in 10^−11^ M, 1.21±0.07 in 10^−8^ M, and 1.04±0.01 in 10^−5^ M dDAVP. The tyrosine phosphorylated protein of 10^−5^ M in 30 kDa was significantly decreased more than 50% compared with control (*p*<0.05) (Fig. 5C and 5D).

### Effect of dDAVP on fertilization and the embryonic development

**Figure 6:**
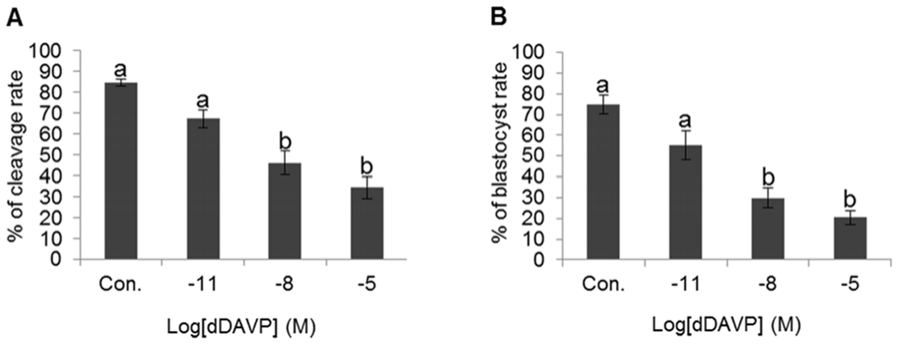
Effect of dDVAP on fertilization and embryo development. (A) Change of cleavage rate in various treatment conditions. (B) Change of blastocyst rate in various treatment conditions. Data represent mean ± SEM, n = 6. Values with different superscripts (^a,b^) were significantly different between control and treatment groups by One-way ANOVA (p<0.05).

The cleavage rate were 84.59±1.65% in control, 67.26±4.33% in 10^−11^ M, 46.08±5.76% in 10^−8^ M, and 34.31±5.31% in 10^−5^ M dDAVP. The cleavage rate of 10^−8^ and 10^−5^ M dDAVP were significantly lowered compared with control (*p*<0.05) (Fig. 6A). Also significant detrimental effects in embryonic development in vitro were observed. The percentage of blastocyst formation was 74.86±4.51% in control, 55.18±7.14% in 10^−11^ M, 29.87±4.78% in 10^−8^ M, and 20.47±3.3% in 10^−5^ M dDAVP. Significantly decreased embryo development were found in 10^−8^ and 10^−5^ M dDAVP (*p*<0.05) (Fig. 6B). These effects were dose-dependent (Fig. 6).

## Discussion

In previous studies, VP has been implicated in stimulating contractile activity of the male reproductive tract [2], [3]. Contractions of seminiferous tubules and epididymal duct walls promote spermiation and sperm transfer, and the contractions are thought to be stimulated by the related peptides VP [3]. The lack of a change in VP concentration with puberty and the absence of any evidence to support local synthesis in sheep epididymis do not support a role in any paracrine or juxtacrine role in the epididymis [27]. Also, VP modulates ion transport and accumulates cytosolic cAMP in vas difference epithelial cells [8]. The VP concentration measured in seminal plasma, 1.84±1.23 pg/ml, is the same as the concentration in blood [28]. While many experiments have addressed the effects of VP in the body, the influence of VP on male fertility has hitherto been unknown.

The present study characterized the effects of VP on sperm function. The presence of AVPR2 in mouse spermatozoa was demonstrated using immune localization. Interestingly, immunofluorescence assays localized AVPR2 exclusively at the tail mid-piece of spermatozoa from the caput epidydimis while positive immunostaining for AVPR2 was detected on the acrosome and the mid-piece in spermatozoa from the cauda epidydimis. It seems that AVPR2 in the acrosome region of spermatozoa develops during sperm maturation in the epidydimis. Although the significance related with reproductive process of this receptor is not known for certain, it is tempting to speculate that the presence of AVPR2 in the head is evidence of possible involvement in the acrosome reaction, and its presence in the tail might be important for the regulation of sperm motility. Therefore, a role of AVPR2 and VP in the events leading to the capacity of spermatozoa to fertilize (or not) the oocyte can be hypothesized. Also, VP modulates ion transport in vas difference epithelial cells [8]. Therefore, AVPR2 and VP may also have highly specialized roles in sperm function, especially related with fertility.

Sperm motility is an important feature and is the most reliable actual predictor of male factor infertility. Presently, motility was evaluated using CASA. The change of motility with VP in a dose-dependent fashion was significant. A high concentration of VP decreased sperm motility. This result implies that VP is detrimental to sperm motility when the spermatozoa undergo capacitation. In agreement with our result, two studies reported that the neutrohormones, oxytocin and VP, significantly decrease the percentage of motility in spermatozoa from human and mouse [29]. However, the tested VP concentrations in previous studies and our study were much higher than the VP concentration actually present in seminal plasma and blood.

For capacitation, extracellular Ca^2+^ flows into spermatozoa because the increase of [Ca^2+^]_i_ is necessary for capacitation and is controlled by Ca^2+^-ATPase [30], [31], [32], [33] with a resulting increase in cAMP concentration [34]. Capacitation leads to acrosome reaction, change of sperm motility, and tyrosine phosphorylation for fertilization by protein kinase A [35], [36]. However, after completion of sperm morphology for sperm-oocyte fusion, [Ca^2+^]_i_ decreases because acrosome reacted spermatozoa release Ca^2+^ from inner cells [21]. Also, the concentration of intracellular cation during capacitation and acrosome reaction increases and intracellular H^+^ is released to extracellular cells. These observations strongly suggest there is an increased [pH]_i_ [36], [37]. [pH]_i_ of spermatozoa in the cauda epididymis is slightly acidic. However, the [pH]_i_ during capacitation shifts to an alkaline level. Also, alkalization that increases [pH]_i_ during capacitation is required for complete capacitation [38], [39], [40].

Presently, according to the data of [pH]_i_ and [Ca^2+^]_i_ by BCECF AM and Fura-2 AM, VP decreased [pH]_i_ and increased [Ca^2+^]_i_ in dose-dependent manners. Especially, higher concentration of treatment group were significantly increased [Ca^2+^]_i_. The results offer functional evidence that VP stimulates ion transport across the sperm membrane through interactions with AVPR2. Also, VP appears to act through AVPR2 to increase [Ca^2+^]_i_.

These observations advance the understanding of sperm physiology as it participates in normal reproductive function. Taken together, these observations strongly suggest that spermatozoa are acutely modulated by VP, in addition to locally released neurotransmitters, that transport defects due to over- or underexpression of AVPR2 and/or AVPR2 mutation, and that aberrant concentration of VP in semen or the male reproductive tract may contribute to male infertility.

To fertilize oocytes in the female reproductive tract and in vitro, the mammalian spermatozoa must undergo the capacitation process that is a prerequisite for the acrosome reaction.

Presently, we confirmed that effect of VP on capacitation status in spermatozoa by CTC staining. VP was significantly decreased in capacitation and the acrosome reaction; especially, high concentrations of VP potently inhibited the acrosome reaction. PKA activity affects directly tyrosine phosphorylation. Tyrosine phosphorylation is regulated PKA in capacitation. Protein tyrosine phosphorylation has an important role in the regulation of processes such as sperm maturation, motility, hyperactivation, cell recognition, and the acrosome reaction [41], [42], [43], all of which are essential for fertilization to occur. Specifically, the change in tyrosine phosphorylation can be monitored using Western blot analysis with α-PY antibodies [44]. Western blotting has confirmed the specificity of PY20 antibody and showed the presence of PY20 in mouse spermatozoa. And PKA activity was confirmed with anti Phospho-PKA antibody [45]. In the present study, five clear Phospho-PKA substrates bands of different sizes were classified in whole range bands. High concentration of VP was significant decrease PKA substrates of approximately 55, 23 and 22 kDa. Also, all bands showed decreased tyrosine phosphorylation in a VP dose-dependent manner. However, only the band of approximately 30 kDa was significantly altered. These data show that AVPR2 is exposed to high concentrations of VP that inhibit PKA activity, tyrosine phosphorylation and capacitation. On the basis of these observations, we suggest that VP effectively inhibits capacitation and the acrosome reaction.

Finally, we evaluated the VP effect on fertilization and embryonic development using in vitro fertilization system. Fertilization and embryonic development were decreased by VP in a dose-dependent fashion. Especially high concentration of VP was significantly decreased fertilization and embryo development rate. Thus, a high concentration of VP affects the sperm-oocyte interaction and embryo development. Since VP is a specific agonist of AVPR2 activation, strongly activated AVPR2 is detrimental to normal sperm function. It is important to point out that in all our experiments, dose-dependent responses were observed: in fact, all conditions were inhibitory.

Taken together, our data have shown that, in mouse spermatozoa, VP stimulates ion transport across sperm membrane through interactions with AVPR2. VP effectively inhibits sperm functions (i.e., the percentage of motility, capacitation status, and protein tyrosine phosphorylation). VP has a detrimental effect in both fertilization and embryonic development, suggesting its critical role in the acquisition of fertilizing ability of mouse spermatozoa. These revelations will enable further elucidation of the molecular mechanisms involved in this particular condition and might shed further light on key sperm proteins involved in capacitation and fertilization. It is also an important prerequisite to the development of diagnostic test to identify affected men in a clinical environment, and in the future might also be applied to the development of novel methods of male targeted contraception.
